# Nurse staffing, nursing assistants and hospital mortality: retrospective longitudinal cohort study

**DOI:** 10.1136/bmjqs-2018-008043

**Published:** 2018-12-04

**Authors:** Peter Griffiths, Antonello Maruotti, Alejandra Recio Saucedo, Oliver C Redfern, Jane E Ball, Jim Briggs, Chiara Dall'Ora, Paul E Schmidt, Gary B Smith

**Affiliations:** 1 Faculty of Environmental and Life Sciences, University of Southampton, Southampton, UK; 2 Department of Learning, Informatics, Management and Ethics, Karolinska Institutet, Stockholm, Sweden; 3 National Institute for Health Research Collaboration for Leadership in Applied Health Research and Care (NIHR CLAHRC) Wessex, Southampton, UK; 4 Dipartimento di Scienze Economiche, Politiche e delle Lingue Moderne, Libera Universita Maria Santissima Assunta, Roma, Italy; 5 Centre for Healthcare Modelling and Informatics Portsmouth, University of Portsmouth, Portsmouth, UK; 6 Medical Sciences Division, University of Oxford Nuffield Department of Clinical Neurosciences, Oxford, UK; 7 Acute Medicine Unit, Portsmouth Hospitals NHS Trust, Portsmouth, UK; 8 Faculty of Health and Social Sciences, University of Bournemouth, Bournemouth, UK

**Keywords:** health services research, mortality (standardized mortality ratios), nurses, health policy, patient safety

## Abstract

**Objective:**

To determine the association between daily levels of registered nurse (RN) and nursing assistant staffing and hospital mortality.

**Design:**

This is a retrospective longitudinal observational study using routinely collected data. We used multilevel/hierarchical mixed-effects regression models to explore the association between patient outcomes and daily variation in RN and nursing assistant staffing, measured as hours per patient per day relative to ward mean. Analyses were controlled for ward and patient risk.

**Participants:**

138 133 adult patients spending >1 days on general wards between 1 April 2012 and 31 March 2015.

**Outcomes:**

In-hospital deaths.

**Results:**

Hospital mortality was 4.1%. The hazard of death was increased by 3% for every day a patient experienced RN staffing below ward mean (adjusted HR (aHR) 1.03, 95% CI 1.01 to 1.05). Relative to ward mean, each additional hour of RN care available over the first 5 days of a patient’s stay was associated with 3% reduction in the hazard of death (aHR 0.97, 95% CI 0.94 to 1.0). Days where admissions per RN exceeded 125% of the ward mean were associated with an increased hazard of death (aHR 1.05, 95% CI 1.01 1.09). Although low nursing assistant staffing was associated with increases in mortality, high nursing assistant staffing was also associated with increased mortality.

**Conclusion:**

Lower RN staffing and higher levels of admissions per RN are associated with increased risk of death during an admission to hospital. These findings highlight the possible consequences of reduced nurse staffing and do not give support to policies that encourage the use of nursing assistants to compensate for shortages of RNs.

## Introduction

In common with health services in many countries, hospitals in the UK find it difficult to fully staff hospital wards. In the face of budgetary constraints and increasing demand for healthcare, there are persistent and growing shortages of registered nurses (RNs).^1^ Many hospitals rely on unregistered nursing assistants to deliver a substantial proportion of ‘hands on’ care. The proportion of fully trained RNs delivering care on hospital wards in England is already among the lowest in Europe.^2^ Recruitment and retention difficulties, combined with ongoing government austerity measures, are set to increase pressure to reduce both the absolute numbers of staff deployed on the wards and the number of RNs relative to the number of nursing assistants.^1 3 4^ However, both reducing the RN workforce and substitution of nursing assistants for RNs have been questioned on the grounds of adverse effects on patient safety.^5 6^ In this paper we explore how variation in the size and composition of the nursing workforce may influence mortality rates in an English National Health Service (NHS) hospital.

In England, a series of inquiries into poor care and adverse outcomes experienced by patients in Stafford Hospital from 2005 to 2009 drew attention to deficiencies in nurse staffing. Following this, the National Institute for Health and Care Excellence (NICE) was commissioned to develop guidance on ‘safe nurse staffing’ in adult inpatient wards. As part of this process NICE commissioned reviews of evidence. Despite the many studies demonstrating hospital-level associations between lower RN staffing levels or dilute skill-mix and adverse patient outcomes, including an increased risk of death,^7^ when NICE published its guidance in 2014, it concluded that:

Insufficient evidence is available about the relationship between staffing, ward-level factors and outcomes. (NICE, p35)^8^

A key factor limiting the extent to which existing research can be used as the basis for informing staffing guidelines is that most studies are cross-sectional, with outcomes and nurse staffing levels measured at the hospital level and averaged over time.^7^ In a notable exception to this pattern, Needleman *et* *al* 9 found that there was a 2% increase in the hazard of death for every occasion when patients were exposed to RN staffing substantially below the level that was planned for a shift during their hospital stay. While this study demonstrated a prospective association between low staffing and death, results were derived from a single US hospital, and the study focused on RN staffing levels only, without examining the contribution of nursing assistants. A more recent study in four Finnish hospitals also demonstrated an association between daily workload per nurse and adverse outcomes, but again did not address composition of the nursing team.^10^

Since European hospitals typically have fewer RNs and a higher proportion of nursing assistants in the nursing care workforce than in the USA,^5^ it is particularly important to consider the potential contributions of assistant staff to maintaining safety. In the current study, we aim to determine whether patients who were exposed to periods of low staffing by RNs and nursing assistants during a stay on general wards in a large general hospital in England experienced an increased risk of death.

## Methods

This was a retrospective longitudinal observational study using routinely collected data about staffing and all patients who spent time on 32 general adult medical and surgical wards (see online supplementary materials) of a large acute care general hospital NHS Trust in the South of England from April 2012 to March 2015.

10.1136/bmjqs-2018-008043.supp1 Supplementary data

### Data sources

Nurse staffing data were extracted from an electronic rostering system, which contained records of shifts worked, location, hours and grade for all nurses employed by the hospital. Temporary staffing deployed on the wards was derived from a database recording bank (extracontractual work by staff employed by the Trust) and agency (staff employed through an external agency) shifts. Both databases were subject to extensive checking and validation as they were directly used to manage staff working patterns and payments. In total, we identified 538 238 shifts worked by RNs (fully qualified nurses on the Nursing and Midwifery Council Register with university diploma or degree-level qualification or equivalent) or nursing assistants (nursing care assistant personnel with no formal training requirements or registration, typically employed in roles described as healthcare assistants or support workers in NHS pay bands 2–4).

Patient data were extracted from the patient administration system and an electronic system used routinely to record patient vital signs (Vitalpac). These data consisted of the following:

- Patient demographic and diagnostic data, including acute diagnosis and comorbidities.
- National Early Warning Score (NEWS) on admission.
- Transfers within the hospital, used to determine the wards where a patient received care and to link patients to staffing data.

Patient data were in turn used to determine the number of patients on each ward and the number of new admissions to the ward for each day. In total, we identified 148 994 patients who spent one or more days on eligible wards in the 3 years of the study.

Our study required that these data sources were linked for analysis purposes. Because shift patterns varied substantially between and within wards, with a mixture of traditional (8 hours) and long (12 hours) shifts, we considered staffing levels in terms of hours per patient day for each staff group. As the calculation of this variable was derived from nursing hours worked and the number of patients on that ward, we had to remove days from the study where we could not reliably match the two. From a theoretical maximum of 35 040 ward days (365 days × 3 years × 32 wards), there were 1822 days on which no patients were recorded on study wards because of ward closures. Of the remaining 33 218 days, 2236 (6.7%) were either unavailable or excluded for other reasons, including days immediately preceding/following ward closure or opening, where extremely low values for the patient population resulted in extremely high nursing hours per patient day. In these cases, the relevant days were excluded from the study when the patient population fell below 25% of the median. In some cases, we could not reliably match the ward codes used for patients with those used for staff. As an example, a ‘ward’ could move from one physical location to another (sometimes occupying part of another existing ward), resulting in situations where patients were coded to the new ward (based on physical location), while the electronic rostering system still recorded staff on the ‘old’ ward. We retained patients where we had at least 1 day of valid staffing measurement. After linking to staffing data, the study sample comprised 138 133 admissions (93% of all potentially eligible admissions).

The study used patient and staff data that were not provided with explicit consent for research purposes. No sensitive data which might aid identification of individuals (eg, postcode area) were transferred to the research team, and all individual identifiers were pseudonymised, meaning that individuals could be linked across data sets but the research team had no way of linking to original identifiers in source files.

### Measures

The primary outcome was death in hospital during the admission, with death within the first 30 days of admission as a secondary outcome. For each admission, we used diagnostic and demographic factors (including diagnosis, age and comorbidity) to calculate a predicted risk of death using coefficients derived from the Summary Hospital Mortality Indicator (SHMI) model.^11^ We used the June 2015 model, which was developed from data on all acute admissions in England for the previous 3 years, approximately coinciding with the study period.^12^ This approach allowed us to estimate the risk associated with a wide range of diagnostic groups (including the effect of interaction between diagnosis and age, comorbidity and admission route) derived from a national population, whereas directly adjusting for mortality risk using only local data would inevitably mean that the risks associated with some diagnoses could not be accurately estimated due to small sample sizes. Because the SHMI risk score reflects the average risk for patients admitted with a given set of characteristics, we also used the patient’s first early warning score, calculated from the first recorded set of vital signs using NEWS, which reflects the patient’s acuity on or near the point of admission. NEWS has been shown to be highly accurate in predicting death in hospital (area under the receiver operating characteristics curve for death within 24 hours: 0.89).^13^ As this measure is taken at the beginning of a patient’s stay, it is largely independent of any effects that may result from variation in nurse staffing, whereas subsequent scores may be influenced by the care received.

Staffing levels were measured as hours per patient per day for RNs and unregistered nursing assistants, with variables normalised as absolute deviations from the mean for each ward, reflecting the different staffing requirements on each ward. The mean staffing for each ward corresponded closely with the hours per patient day estimated from planned staffing levels (establishments), which were determined by a widely used staffing methodology, the Safer Nursing Care Tool^14^ (online supplementary table a).

We considered staffing as both a categorical variable (days with staffing below the ward mean hours per patient day) and as a continuous variable (daily hours above/below ward mean) in separate analyses. Because the level of nursing work is also likely to be influenced by the turnover of patients caused by admissions and discharges,^9^ we calculated a variable to indicate days of unusually high patient turnover for the ward relative to the available workforce. Since ward establishments are set to accommodate typical admission levels, we identified days when admissions per nurse exceeded 125% of the mean for that ward—reflecting a mismatch between demand and available staff, resulting from either a low number of staff or a high number of admissions.

### Analysis

We used multilevel/hierarchical mixed-effects survival models to explore the association between staffing levels and death. A proportional hazard model was specified. In addition to the staffing level variable, all models included variables to control for patient risk (SHMI risk score, first NEWS and emergency admission), patient turnover (admissions per RN/nursing assistant above 125% of mean) and ward as a random effect. Survival models consider the time to event (in this case death), and the resulting HR considers the relative rate of death over a period of time.^15^ Because staffing levels vary both between and within wards and patients move from ward to ward, we considered staffing levels as time-varying covariates in a repeated-measures framework, with all staffing level variables normalised relative to the mean for the current ward. Thus our estimates of the effect of staffing reflect variation within wards rather than variation between wards. Variation between wards may simply reflect an assessment of different levels of care required. RN and nursing assistant staffing levels were expressed as a cumulative sum of exposure. Staffing on wards other than general wards (eg, intensive care unit) was not considered and so did not contribute to the cumulative sum. We explored non-linear effects of staffing levels by including quadratic and cubic terms in models. Relative improvements in model fit were assessed by looking for reductions in the Akaike information criterion and Bayesian information criterion. An exponential distribution was assumed for the baseline hazard function, selected as it provided superior model fit to the Cox proportional hazards, where no distribution is assumed. Analyses were undertaken using the Stata Statistical Software: Release V.14 using the xtstreg command.

Our primary analysis considered staffing during the first 5 days of stay, reflecting the majority of the stay for the majority of patients, but we also undertook secondary analysis considering all days of the stay. The analysis thus focuses on the period of hospital stay when the patient is most likely to be acutely ill, while still including staffing levels experienced by the majority of patients for the majority of their stay (median stay is less than 3 days). This approach to modelling reflects that taken by Needleman and colleagues.^9^

## Results

### Patient and staffing characteristics

The majority of patients were admitted as emergencies (79%). The mean age of admitted patients was 67 years, with 14% aged ≥85 years. While 50% had no comorbidities (Charlson Comorbidity Index (CMI) 0), 35% of patients had a CMI score of ≥5 (table 1). Overall, 5662 patients died (4.1%). One hundred and thirty-seven different diagnostic groups were represented as the main diagnosis associated with the admission, based on the diagnostic groups used to calculate SHMI.^11^ Pneumonia was the single most common diagnosis (4.2%), with the most common 15 diagnostic groups accounting for 38% of all admissions. The diagnostic group associated with most deaths was also pneumonia (21% of all deaths). The mean length of stay was 6.8 days (median 2.7).

**Table 1:** Patient characteristics

	n (%)	Mean (SD)	Median (range)
All patients	138 133 (100)		
Emergency admissions	108 865 (79)		
Elective	29 268 (21)		
Male	64 596 (47)		
Female	73 537 (53)		
Age		62.93 (20.61)	66.64 (16.03–106.14)
Under 65	64 984 (47)		
65 to <75	25 223 (18)		
75 to <85	28 316 (21)		
85+	19 610 (14)		
Charlson Comorbidity Index*		6.08 (84.26)	3 (0–98)
0	68 682 (50)		
1–2	231 (0.2)		
3–4	20 385 (15)		
5+	48 663 (35)		
SHMI risk		0.06 (0.10)	0.01 (0.00–0.85)
First NEWS		1.73 (2.03)	1 (0–19)
Low risk (NEWS≤2)	102 674 (74)		
Medium risk (NEWS≥3 and NEWS≤5)	27 409 (20)		
High risk (NEWS≥6)	8050 (6)		
Length of stay		6.81 (12.63)	2.73 (0.15–933.33)

*Charlson. *et* *al* 30

CMI, Charlson Comorbidity Index; NEWS, National Early Warning Score; SHMI, Summary Hospital Mortality Indicator.

Across all wards the mean staffing levels were 4.75 RN hours per patient per day and 2.99 nursing assistant hours per patient per day. The mean skill-mix was 61% RN. Staffing levels varied considerably both between and within wards (online supplementary table a). The mean RN hours per patient per day varied from 2.91 (a general medical respiratory ward) to 9.61 (renal high care). Skill-mix (proportion of RNs) varied from 86% (renal ward) to 46% (medical general ward). The SD for variation of staffing within wards was, on average, 18% of the mean for RN hours per patient per day and 29% of the mean for nursing assistant hours per patient per day. Over the first 5 days of their hospital stay, patients experienced a mean of 1.93 days of low RN staffing and 1.94 days of low nursing assistant staffing, with a mean cumulative shortfall of 0.39 RN hours per patient and 0.25 nursing assistant hours per patient (online supplementary table b). Admissions above 125% of the ward mean occurred on around a quarter of all days (27% for admissions per RN and 26% for admissions per nursing assistant), with patients exposed to a mean of 0.88 days of high admissions per RN and 0.91 days of high admissions per nursing assistant during their first 5 days (online supplementary table c).

### Staffing, turnover and mortality

For each day that a patient spent on a ward with RN staffing below the mean for that ward, the hazard of death was increased by 3% (adjusted HR (aHR) 1.03, 95% CI 1.01 to 1.06). Each day of exposure to nursing assistant staffing below the mean was associated with a 4% increase in the hazard of death (aHR 1.04, 95% CI 1.02 to 1.07). When patients were on wards where admissions per RN exceeded 125% of the mean for that ward, the hazard of death was increased by 5% (aHR 1.05, 95% CI 1.01 to 1.09), but there was no significant association between admissions per nursing assistant and death (table 2).

**Table 2*†:** Hazard of death associated with low staffing levels during the first 5 days (adjusted and unadjusted)

Staffing exposure (per day over the first 5 days)	HR*	P values	95% CI	Adjusted HR†	P values	95% CI
RN staffing below ward mean	1.03	0.009	1.01 to 1.06	1.03	0.009	1.01 to 1.06
NA staffing below ward mean	1.04	<0.001	1.02 to 1.07	1.04	<0.001	1.02 to 1.07
Admissions per RN >125% of ward mean	1.05	0.024	1.01 to 1.09	1.05	0.024	1.01 to 1.09
Admissions per NA >125% of ward mean	1.00	0.873	0.96 to 1.04	1.00	0.873	0.96 to 1.04
NEWS on admission	1.30	<0.001	1.28 to 1.31	1.26	<0.001	1.24 to 1.27
SHMI risk score	1.95	<0.001	1.90 to 2.00	1.87	<0.001	1.81 to 1.92
Emergency	3.89	<0.001	3.16 to 4.79	1.24	0.085	0.97 to 1.60

Df 41; AIC 61 889.87; BIC 62 376.13.

Unconditional model adjusting for ward (random effect) only.

Adjusted for ward, SHMI risk score, NEWS on admission and mode of admission (emergency vs elective). For full model see online supplementary table a.

AIC, Akaike information criterion; BIC, Bayesian information criterion; NA, nursing assistant; NEWS, National Early Warning Score; RN, registered nurse; SHMI, Summary Hospital Mortality Indicator.

When considering deaths within 30 days of admission only, the model coefficients were almost identical. We also considered deaths within 5 and 10 days of admission only, which yielded a similar pattern of results, although for deaths within 5 days the effect of low RN staffing was greater and the effect of low nursing assistant staffing smaller and non-significant (online supplementary tables e-g). Similarly, restricting the model to emergency admissions only had no effect on the coefficients for the staffing variables. Models including exposure to low staffing on all days of the hospital stay showed significant adverse effects from low RN staffing only, although the coefficients were lower (aHR 1.01 per day), a likely product of increased risk being primarily associated with days early in the stay (online supplementary tables h-i).

Adding variables to account for weekend admissions or weekend stay, periods where low staffing by other professions might co-occur with low staffing by nurses, did not substantially alter the coefficients or the substantive statistical conclusions (online supplementary tables j-k). We considered the possibility that low staffing might co-occur with seasonal infections by examining the frequency of influenza as an admission diagnosis among patients who died. Influenza occurs marginally less often among those exposed to low staffing compared with those not exposed (0.5% vs 0.8% for low staffing on first day, 0.5% vs 1.3% for low staffing on any day; online supplementary table l). We looked for seasonal trends in staffing and found no clear pattern, although RN staffing was higher in winter months compared with summer months, whereas nursing assistant staffing was lower at the beginning of the year (late winter) compared with summer months and early winter (online supplementary figures a,b). Finally we calculated the association between staffing levels relative to ward mean and patient risk (SHMI and first NEWS) and found no significant associations (online supplementary tables m-r).

In order to further quantify the effect of variation in staffing levels, we explored the effects of absolute variations by calculating the cumulative sum of staffing in hours per patient per day relative to the mean for the ward for each patient for each of the first 5 days. This gives an indication of the average staffing experienced by the patient, relative to what was normal for each ward (table 3). Every additional RN hour per patient was associated with a 3% reduction in the hazard of death (aHR 0.97, 95% CI 0.94 to 1.00). However, additional nursing assistant hours were not significantly associated with a reduced hazard of death (aHR 1.01, 95% CI 0.98 to 1.04). The results were similar when considering only the cumulative sum of hours below the mean (see online supplementary tables s-t).

**Table 3:** Hazard of death associated with cumulative hours above or below the mean during the first 5 days (linear and non-linear models)

Staffing exposure (cumulative sum over the first 5 days)	Adjusted HR	P values	95% CI
Linear model			
RN hours	0.97	0.023	0.94 to 1.00
NA hours	1.01	0.394	0.98 to 1.04
Df 41; AIC 61 919.4; BIC 42 405.66			
Non-linear model			
RN hours	0.98	0.200	0.95 to 1.01
RN hours^2^	1.00	0.621	0.99 to 1.01
RN hours^3^	1.00	0.930	1.00 to 1.00
NA hours	1.00	0.835	0.97 to 1.04
NA hours^2^	1.01	0.014	1.00 to 1.02
NA hours^3^	1.00	0.061	1.00 to 1.00

Df 45; AIC 61 920.19; BIC 62 453.88.

All models include control for turnover (admissions per nurse), patient risk (SHMI), first NEWS, emergency admission and ward (for full model see online supplementary tables g,h).

AIC, Akaike information criterion; BIC, Bayesian information criterion; NA, nursing assistant; NEWS, National Early Warning Score; RN, registered nurse; SHMI, Summary Hospital Mortality Indicator.

**Figure 1:**
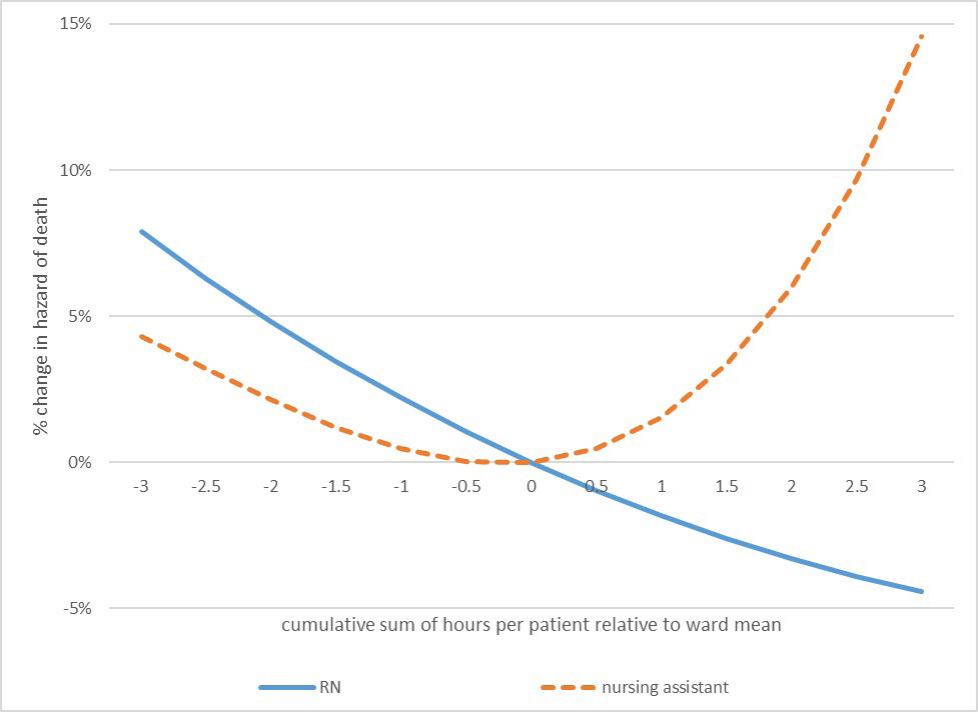
Non-linear effects of nurse staffing. Change in the hazard of death associated with variation in staffing levels, relative to the mean. RN, registered nurse.

The contrast between analyses treating staffing as a categorical variable and treating it as a continuous variable, particularly in relation to nursing assistant staffing levels, raises the possibility of a non-linear effect. We explored this by introducing quadratic and cubic terms for the staffing variables into the models. The coefficients are reported in table 3. The quadratic term for nursing assistant staffing was significant and the cubic term neared significance, indicating a non-linear relationship. In order to understand the non-linear relationship, we plotted the curves based on these coefficients. The hazard of death was increased when patients were exposed to either more than average or less than average nursing assistant hours over the course of their stay, while the relationship between RN staffing and the hazard of death appears to be linear (figure 1). We also considered whether there was evidence that variation in levels of nursing assistant staffing could alter the effect of RN staffing (or vice versa) by introducing an interaction term into the model. There was no significant interaction between RN hours and nursing assistant hours (online supplementary table v).

## Discussion

When patients experienced days where staffing levels fell below the ward’s mean, their risk of death was increased. While the relationship appeared to be linear for RN staffing levels, there was evidence that the risk of death increased as patients were exposed to levels of nursing assistant staffing that were either above or below the mean. While our study largely confirms existing findings about the association between RN staffing and outcomes, it provides important additional information. First, the association is demonstrated at a patient level with a longitudinal association between variation in staffing and outcome, providing important confirmation that the hospital-level cross-sectional associations reflect individual patient exposures. Second, we have demonstrated that the relationship appears to be linear with no threshold effect over the range of variation that we observed. Although this is the first study to show a significant harmful effect from low nursing assistant staffing, we also show a potential harm when staffing from this group is above the mean.

The NHS has begun to use a standard metric of ‘care hours per patient day’ to provide a comparison of staffing levels between hospitals.^3^ This measure combines registered nursing and nursing care assistant hours to give a single measure. Our findings clearly show that a measure combining these two staff groups in this way is unwarranted. Cross-sectional studies have found that lower skill-mix has been associated with worse outcomes, including mortality (eg, refs^5 16 17^). However, if only examining the proportion of RNs in the workforce, the independent contribution of each staff group cannot be properly determined. Most studies that have considered both RNs and other nursing staff groups as independent predictors have found that having more assistant nursing personnel was not associated with mortality or was associated with increased mortality at a hospital level.^18–21^ One quasi-experimental study found mixed effects on mortality and adverse effects on several other outcomes from adding assistants to the workforce.^22^

Although our findings lend no support to a policy of compensating for deficits in the RN workforce by employing more nursing assistants, these results do show that an adequate number of assistants is important for maintaining patient safety. Findings of some studies show a net negative effect from adding any assistant staff at any given level of RN staffing, because of the reduction in skill-mix.^5^ Our results show that there may be an optimal level of assistant staffing. The mean staffing levels on the wards we studied corresponded closely to the staffing establishments (planned staffing levels), which were determined by a widely used staffing methodology, the Safer Nursing Care Tool.^14^ When assistant staffing was close to this level, the hazard of death was reduced. The mechanism by which adding further assistants above this level can lead to worse outcomes requires further exploration. Increases in assistant staffing levels in response to increases in patient dependency is a possible explanation, although we found no association between our measures of mortality risk and assistant staffing levels, which makes this unlikely. It may be that the presence of additional direct care staff creates a division of labour that means RNs spend less time with patients, reducing opportunities for ongoing monitoring, assessment and evaluation outside scheduled observations.^22^

While nursing assistant staffing above the mean was associated with no additional benefit (indeed it was associated with increases in mortality), the same was not the case for RN staffing. The relationship between RN staffing hours and mortality appeared to be linear. This is in itself a surprising finding. While many previous studies have reported a linear coefficient, few have directly explored whether the relationship was, in fact, linear, although a meta-analysis of North American studies found a curvilinear relationship between staffing and outcomes.^23^ In that study, the incremental benefits of adding more staff reduced at higher staffing levels. Although our findings cannot be directly compared with hospital-level studies, which provide hospital-wide averages, nurse staffing levels in English hospitals are generally lower than those found in many other European countries and the USA, and typical patient to nurse ratios are higher than agreed or mandatory levels in other countries.^2 5 24^ Consequently, it may be that RN staffing in our study hospital does not reach levels at which incremental benefits diminish.

The average overall staffing (nursing assistant+RN hours) in this study, approximately 7.75 hours per patient per day, is typical of NHS general hospitals, although it falls at the lower end of those reported in the Carter Review of productivity in the NHS (where the range was from 6.3 to 15.48).^3^ An additional RN hour per patient day would represent a staffing uplift of 13%, equivalent to one additional RN per shift on a 24-bed ward (excluding any shift overlaps). Patients experiencing the median length of stay would have a cumulative increase of 2.7 nursing hours, associated with a reduction in the hazard of death of 8%.

Patient turnover has been identified as an important influence on nurse staffing requirements in previous research. Needleman and colleagues^9^ found that patients exposed to shifts with high turnover experienced a 7% increase in the hazard of death.^9^ Our findings suggest that the association is specific to RN staffing, although our threshold (above 125% of the mean) was not subjected to sensitivity testing. As the work of assessing and planning care for new patients is an RN responsibility, the additional workload is likely to fall primarily on them. While we did not consider discharges directly, these are likely to be highly correlated with admissions in the face of high bed occupancy. Again, preparation for discharge is likely to generate significant additional work for RNs. Workload and productivity measures that focus solely on patient numbers may significantly underestimate the registered nursing workload, particularly if the length of stay shortens and patient turnover increases. The significance of patient turnover as a workload variable in addition to patient census and acuity/dependency merits more attention in future research.

The findings of this paper suggest potential benefits from increasing the availability of RNs on acute hospital wards. However, in England, RN shortages look set to continue in the short term. These findings show the importance of a policy response in order to rectify the shortage and to retain existing RNs within the workforce. Health Education England, which has a responsibility for workforce planning and training staff for the NHS, has recently announced plans to increase RN training posts and has shown a renewed interest in programmes that could improve retention of nurses such as Magnet accreditation.^25 26^ These findings indicate that RN shortages are unlikely to be remedied by increasing the numbers of lesser trained nursing staff in the workforce.

### Strengths and limitations of this study

While our study was able to overcome many limitations of previous research, it remains observational. Causal inference does not follow directly from the observed associations, although the longitudinal design with staffing exposures measured at a patient level eliminates many plausible alternative explanations making a causal interpretation much more likely.^27^ Additionally, there is a growing body of evidence supporting a causal mechanism: linking low nurse staffing to increased mortality through a reduced capacity to observe and intervene to prevent deterioration.^28 29^

Our study has a number of strengths. Although our study was in a single site, this does mean that our findings are not the result of a ‘hospital effect’, whereby the observed associations between nurse staffing and outcomes could arise because hospitals with more resources in general also have more nurses.^27^ It is the first study to consider the longitudinal relationship with RN and nursing assistant staffing independently. We have assessed the staffing levels experienced by individual patients and shown that exposure to low staffing precedes increased risk of death. The use of survival analysis means that we have considered time to event, but our data are censored at the point of discharge and our analysis assumes that those discharged early have the same risk as those discharged late. It is possible that the large proportion of patients with no comorbidities in our sample is a result of undercoding, but the distribution of recorded comorbidities resembles the sample on which the SHMI risk model was validated.^11^ In addition to the SHMI risk score, our models included a measure of acuity as a covariate, which is also a good predictor of mortality risk, and so we are confident that we have accounted for patient-level risk factors as far as possible.

Results based on hours relative to the mean measure are not subject to bias arising from patients with longer stays experiencing more low staffing. Our measure of staffing is determined relative to the average for each ward rather than an absolute measure, and so it accounts for different requirements for care in different wards. In simple terms this means that our analysis reflects association between mortality and variation in nurse staffing within each ward, rather than between wards. While average ward staffing levels corresponded closely with the measured staffing requirements, any effect from variation in average staffing levels between wards is not reflected in these analyses. Nonetheless, we cannot exclude all possible sources of endogeneity.^7^

## Conclusion

These findings show the potential consequences of shortages of RNs in terms of the negative impact on patient safety. While nursing care assistants also have an important part to play in maintaining the safety of hospital wards, they cannot act as substitutes for RNs. When assessing staffing requirements or making comparisons, RN and assistant hours should not be treated as equivalent. Strategies to improve the supply of RNs are required. The adverse consequences of RN shortages are unlikely to be remedied by increasing the numbers of lesser trained nursing staff in the workforce.
