# Supplementary material for: Nurse staffing, nursing assistants and hospital mortality: retrospective longitudinal cohort study
Source: BMJ Qual Saf. 2018 Dec 4;28(8):609–17. doi: 10.1136/bmjqs-2018-008043 (PMC6716358; doi:10.1136/bmjqs-2018-008043)
Supplement: Supplementary data [file bmjqs-2018-008043supp001.pdf]

SUPPLEMENTARY MATERIAL

Included wards

LABEL	Description	Beds
Cancer	Medical / radiotherapy haematology / oncology	40
Med/Surg Cardiac	Medical / Surgical cardiac high care & step down	23
Med-adm	Medical - emergency admissions	58
Med-gastro	Medical - gastro & liver	36
Med-gen1	Medical - general cardiac & gastro	36
Med-gen2	Medical - general	30
Med-gen3	Medical - general	35
Med-OP1	Medical - older people	30
Med-OP2	Medical - older people	20
Med-OP2	Medical - older people	29
Med-OP3	Medical - older people	30
Med-OP4	Medical - older people	21
Med-renal	Medical - renal	24
Med-renal HC	Medical - renal high care / step down	10
Med-resp1	Medical- respiratory high care and step down	40
Med-resp2	Medical- respiratory	36
Med-stroke	Medical - acute stroke	12
Med-Surg	Medical / Surgical elective & investigations	13
Non study	(in regression analyses when patients spent time on non-study wards)	
Rehab-neuro	Rehabilitation - neurological (working age)	13
Rehab-stroke	Rehabilitation - stroke (older people)	26
Surg-adm	Surgical - admissions	28
Surg-el/ortho2	Surgical -elective orthopaedic	36
Surg-em/hip#	Surgical - fracture neck of femur / older people	36
Surg-em/ortho2	Surgical - emergency orthopaedic (spinal)	26
Surg-em/ortho2	Surgical - emergency orthopaedic (head injury)	26
Surg-gen1	Surgical -general urology, vascular, plastic	37
Surg-GI	Surgical - general, upper GI	30
Surg-GI	Surgical - general, colorectal	34
Surg-gynae	Surgical - gynaecological	22
Surg-H&N	Surgical - head & Neck	21
Surg-HC	Surgical - high care, step down / step up	10
Surg-renal	Surgical - renal transplant	14

Note on included wards

We set out to study the effects of staffing on 'general' adult medical and surgical wards. Wards with specialties such as maternity care and paediatrics were excluded because staffing and patient risk profiles are fundamentally diffent. Our operational definition of general wards were those where staffing was determined using a common standard based on the Safer Nursing Care Tool[1]. Some wards that included some high dependency beds were thus included, but ICU was excluded. Patients who spent time in non-study wards were included in the study but only exposures to staffing on the included wards were considered.

Supplementary table a: Nurse and care hours per patient day & skill-mix by ward

Ward*	days of staffing data	RN			NA			All care hours (RN+NA)			Skill-mix (RN/RN+NA)		
		establishment*	mean (SD)		establishment	mean (SD)		establishment	mean (SD)		establishment	mean (SD)	
CANCER	1095	5.49	5.52	(0.64)	2.22	2.25	(0.30)	7.71	8.58	(0.39)	71%	71%	(3%)
MED/SURG	1095	7.61	6.40	(0.69)	0.78	1.40	(0.64)	8.39	7.01	(0.89)	91%	82%	(7%)
MED-ADM	1095	7.19	6.92	(1.08)	2.32	2.72	(0.58)	9.51	6.05	(0.69)	76%	72%	(3%)
MED-GASTRO	1095	3.73	3.49	(0.50)	2.33	3.51	(0.89)	6.06	7.80	(1.27)	62%	50%	(7%)
MED-GEN1	1095	3.37	3.12	(0.45)	2.67	2.92	(0.55)	6.04	7.77	(0.74)	56%	52%	(5%)
MED-GEN2	494	3.99	3.18	(0.56)	3.17	3.78	(0.72)	7.16	6.96	(1.00)	56%	46%	(6%)
MED-GEN3	1046	3.94	3.43	(0.63)	3.36	2.50	(0.59)	7.30	5.93	(0.72)	54%	58%	(6%)
MED-OP1	1040	4.98	4.33	(1.49)	3.89	4.22	(1.41)	8.87	7.35	(1.51)	56%	51%	(7%)
MED-OP2	197	3.92	3.77	(0.75)	3.92	4.21	(0.75)	7.84	7.12	(0.88)	50%	47%	(6%)
MED-OP2	1095	3.55	3.64	(0.45)	3.29	3.33	(0.48)	6.84	8.30	(0.60)	52%	52%	(5%)
MED-OP3	1095	3.72	3.87	(0.64)	3.44	3.56	(0.62)	7.16	5.98	(0.82)	52%	52%	(6%)
MED-OP4	864	3.94	3.81	(0.72)	3.55	4.32	(1.01)	7.49	6.91	(1.51)	53%	47%	(9%)
MED-RENAL	894	8.51	7.30	(1.01)	1.39	1.91	(0.56)	9.90	5.58	(0.74)	86%	79%	(5%)
MED-RENAL HC	1095	10.76	9.61	(1.54)	3.01	2.59	(0.95)	13.77	5.88	(0.77)	78%	79%	(7%)
MED-RESP1	854	5.05	5.19	(0.52)	1.89	2.56	(0.46)	6.94	7.74	(0.66)	73%	67%	(5%)
MED-RESP2	1095	3.31	2.91	(0.48)	2.62	2.95	(0.74)	5.93	5.85	(0.91)	56%	50%	(7%)
MED-STROKE	1027	3.55	3.88	(0.62)	3.31	3.36	(0.65)	6.86	8.99	(0.86)	52%	54%	(6%)
MED-SURG	694	6.75	6.33	(1.50)	2.95	2.79	(0.84)	9.70	8.55	(0.95)	70%	70%	(5%)
REHAB-NEURO	1044	4.89	5.00	(1.06)	3.49	3.99	(1.39)	8.38	7.57	(1.31)	58%	56%	(7%)
REHAB-STROKE	794	3.86	3.57	(0.53)	3.7	4.00	(0.47)	7.56	7.25	(0.61)	51%	47%	(5%)
SURG-ADM	1095	4.1	3.64	(0.75)	1.8	1.96	(0.55)	5.90	7.77	(0.66)	69%	65%	(7%)
SURG-EL/ORTHO2	1083	4.54	4.06	(1.07)	2.36	3.06	(0.72)	6.90	7.98	(0.85)	66%	57%	(5%)
SURG-EM/HIP#	1095	4.09	4.36	(0.88)	2.62	3.92	(0.99)	6.71	6.98	(1.09)	61%	53%	(5%)
SURG-EM/ORTHO2	1017	4.35	4.24	(0.87)	3.31	3.53	(0.92)	7.66	7.43	(1.28)	57%	55%	(7%)
SURG-EM/ORTHO2	1047	4.36	4.04	(0.57)	2.79	3.30	(0.68)	7.15	8.14	(0.85)	61%	55%	(6%)
SURG-GEN1	1095	4.27	3.30	(0.51)	2.37	2.68	(0.42)	6.64	9.12	(0.52)	64%	55%	(5%)
SURG-GI	1095	3.56	3.39	(0.49)	2.56	2.19	(0.34)	6.12	12.20	(0.43)	58%	61%	(5%)
SURG-GI	1095	3.6	3.51	(0.59)	2.4	2.37	(0.39)	6.00	9.22	(0.45)	60%	60%	(5%)
SURG-GYNAE	874	4.48	5.71	(1.66)	1.74	2.87	(1.01)	6.22	9.40	(1.04)	72%	66%	(7%)
SURG-H&N	1092	5.14	4.68	(0.91)	2.48	2.24	(0.77)	7.62	9.64	(0.92)	67%	68%	(8%)
SURG-HC	1095	9.77	8.54	(1.43)	1.7	2.27	(0.84)	11.47	5.59	(1.17)	85%	79%	(7%)
SURG-RENAL	494	7.95	7.11	(1.15)	2.23	2.29	(0.69)	10.18	10.81	(0.84)	78%	76%	(6%)
Mean	968.13	5.07	4.75	(0.84)	2.677	2.99	(0.72)	7.75	7.73	(0.87)	64%	60%	(6%)
*See over for elaboration of ward labels													

Mean RN HPPD was highly correlated with the estimated RN HPPD estimated from the planned establishment (Pearson r 0.97) with average RNHPPD 95% of the establishment level. Similarly, mean NURSING ASSISTANT staffing was closely correlated with the establishment (Pearson r 0.81) although there was more variation, with mean NURSING ASSISTANT staffing 115% of establishment. Establishments were given as staff per shift which could, in some cases, be met by a partial shift to avoid shift overlaps or by either NA or RN and so hours are estimates only. Therefore, the significance of any discrepancy is unclear. For this reason, we relied on means to indicate 'normal' staffing, derived from hours worked.

Supplementary table b: Patient exposure to low staffing (n=138,133)

**RN Hours per patient per day**	**Mode**	**Median**	**Mean**	**Min**	**Max**
Days below mean	0	2	1.93	0	5
Hours below mean	0	0	0.39	0	36
**NA Hours per patient per day**	**Mode**	**Median**	**Mean**	**Min**	**Max**
Days below mean	0	2	1.94	0	5
Hours below mean	0	0	0.25	0	30

Supplementary table c: Admissions per staff member

Ward	days of data (N)	Mean SD		n(%) days 25% above mean	Mean SD		n(%) days 25% above mean
CANCER	1,095	0.78	0.32	263 (24%)	1.94	0.85	281 (26%)
MED/SURG CARDIAC	1,095	1.47	0.64	300 (27%)	8.59	6.33	285 (26%)
MED-ADM	1,095	3.44	0.54	59 (5%)	8.89	1.73	115 (11%)
MED-GASTRO	1,095	1.70	1.01	272 (25%)	1.79	1.20	287 (26%)
MED-GEN1	1,095	1.89	0.78	281 (26%)	2.06	0.91	280 (26%)
MED-GEN2	494	1.30	0.76	143 (29%)	1.11	0.67	142 (29%)
MED-GEN3	1,046	0.92	0.96	238 (23%)	1.31	1.50	233 (22%)
MED-OP1	1,040	1.10	0.56	275 (26%)	1.16	0.64	284 (27%)
MED-OP2	197	0.71	0.67	63 (32%)	0.63	0.58	58 (29%)
MED-OP2	1,095	0.58	0.44	341 (31%)	0.65	0.51	327 (30%)
MED-OP3	1,095	0.51	0.36	344 (31%)	0.56	0.40	352 (32%)
MED-OP4	864	0.57	0.60	252 (29%)	0.62	2.90	198 (23%)
MED-RENAL	894	0.50	0.29	273 (31%)	2.10	1.52	250 (28%)
MED-RENAL HC	1,095	0.41	0.35	375 (34%)	1.75	1.99	245 (22%)
MED-RESP1	854	1.19	0.67	159 (19%)	2.48	1.52	166 (19%)
MED-RESP2	1,095	2.12	1.23	225 (21%)	2.16	1.26	260 (24%)
MED-STROKE	1,027	0.57	0.45	303 (30%)	0.68	0.53	295 (29%)
MED-SURG	694	1.95	1.06	204 (29%)	4.63	2.75	197 (28%)
REHAB-NEURO	1,044	0.15	0.29	308 (30%)	0.20	0.38	308 (30%)
REHAB-STROKE	794	0.4	0.50	259 (33%)	0.37	0.45	255 (32%)
SURG-ADM	1,095	5.45	1.84	213 (19%)	10.41	3.83	236 (22%)
SURG-EL/ORTHO2	1,083	2.21	1.46	413 (38%)	2.94	1.99	412 (38%)
SURG-EM/HIP#	1,095	1.56	0.90	343 (31%)	1.78	1.05	349 (32%)
SURG-EM/ORTHO2	1,017	0.82	0.60	318 (31%)	0.98	0.75	301 (30%)
SURG-EM/ORTHO2	1,047	1.07	0.64	304 (29%)	1.34	0.83	295 (28%)
SURG-GEN1	1,095	1.88	0.81	263 (24%)	2.30	0.95	287 (26%)
SURG-GI	1,095	2.06	0.90	280 (26%)	3.26	1.55	286 (26%)
SURG-GI	1,095	1.45	0.68	289 (26%)	2.18	1.11	302 (28%)
SURG-GYNAE	874	2.26	0.94	225 (26%)	4.69	2.34	223 (26%)
SURG-H&N	1,092	2.24	0.91	289 (26%)	5.54	4.25	249 (23%)
SURG-HC	1,095	0.95	0.51	342 (31%)	4.06	3.17	271 (25%)
SURG-RENAL	494	0.72	0.47	147 (30%)	2.42	1.82	129 (26%)
ALL (ward)	968	1.40	0.72	261 (27%)	2.67	1.63	255 (26%)

Supplementary table d: Days of staffing below the mean during the first five days: hazard of death (full model)

	Haz. Ratio	Std. Err.	p-value	95% Confidence Interval (CI)	
NEWS on admission	1.24	0.007	<0.001	1.23	1.25
SHMI Risk score	1.82	0.025	<0.001	1.78	1.87
Emergency	1.11	0.124	0.365	0.89	1.38
Admissions per RN>125% of ward mean	1.05	0.023	0.024	1.01	1.09
Admissions per NA>125% of ward mean	1.00	0.022	0.873	0.96	1.04
RN staffing below ward mean	1.03	0.012	0.009	1.01	1.06
NA staffing below ward mean	1.04	0.012	<0.001	1.02	1.07
Ward dummy (surg-gynae is reference)	1.00				
Med-gastro	2.70	0.692	<0.001	1.64	4.47
Med-gen1	1.78	0.459	0.025	1.07	2.95
Med/Surg Cardiac	2.17	0.565	0.003	1.30	3.61
Surg-em/ortho2	0.65	0.190	0.143	0.37	1.16
Med-gen2	1.13	0.311	0.656	0.66	1.94
Med-gen3	0.95	0.257	0.847	0.56	1.61
Surg-em/ortho2	0.83	0.246	0.528	0.46	1.48
Surg-el/ortho2	0.76	0.369	0.574	0.29	1.97
Surg-em/hip#	0.43	0.167	0.030	0.20	0.92
Surg-gen1	1.44	0.388	0.173	0.85	2.44
Surg-H&N	0.67	0.217	0.217	0.36	1.26
Surg-GI	1.34	0.368	0.288	0.78	2.29
Surg-GI	1.45	0.389	0.171	0.85	2.45
Med-resp1	2.80	0.704	<0.001	1.71	4.59
Med-resp2	2.34	0.592	0.001	1.43	3.84
Rehab-neuro	0.04	0.040	0.002	0.01	0.29
Med-OP1	2.55	0.649	<0.001	1.55	4.20
Rehab-stroke	0.62	0.174	0.090	0.36	1.08
Med-stroke	2.04	0.519	0.005	1.24	3.36
Cancer	2.66	0.673	<0.001	1.62	4.37
Med-OP2	2.46	0.763	0.004	1.34	4.52
Med-OP2	1.95	0.497	0.009	1.19	3.22
Med-OP3	2.64	0.671	<0.001	1.61	4.35
Med-OP4	2.02	0.530	0.007	1.21	3.38
Med-Surg	2.14	0.630	0.010	1.20	3.81
Med-renal HC	1.81	0.514	0.036	1.04	3.16
Med-renal	1.77	0.477	0.033	1.05	3.01
Surg-renal	1.21	0.386	0.549	0.65	2.26
Med-adm	1.81	0.456	0.019	1.10	2.96
Non study	1.41	0.357	0.180	0.85	2.31
Surg-adm	2.96	0.774	<0.001	1.78	4.95
Surg-HC	2.36	0.695	0.004	1.32	4.20
df 41, AIC 61889.87, BIC 62376.13					

Supplementary table e: Staffing below the mean during the first five days: hazard of death within 30 days

	Haz. Ratio	Std. Err.	p-value	95% Confidence Interval (CI)	
NEWS on admission	1.26	0.007	<0.001	1.24	1.27
SHMI Risk score	1.87	0.027	<0.001	1.81	1.92
Emergency	1.24	0.158	0.085	0.97	1.60
Admissions per RN>125% of ward mean	1.05	0.024	0.044	1.00	1.09
Admissions per NA>125% of ward mean	1.02	0.023	0.443	0.97	1.06
RN staffing below ward mean	1.03	0.013	0.014	1.01	1.06
NA staffing below ward mean	1.04	0.013	0.001	1.02	1.07
(full model includes random effects for ward, omitted for brevity) df 41, AIC 54395.58, BIC 54876.58					

Supplementary table f: Staffing below the mean during the first five days: hazard of death within 10 days

	Haz. Ratio	Std. Err.	p-value	95% Confidence Interval (CI)	
NEWS on admission	1.31	0.01	0.000	1.29	1.33
SHMI Risk score	1.95	0.03	0.000	1.89	2.02
Emergency	1.92	0.36	0.001	1.33	2.78
Admissions per RN>125% of ward mean	1.05	0.03	0.091	0.99	1.11
Admissions per NA>125% of ward mean	1.03	0.03	0.273	0.98	1.09
RN staffing below ward mean	1.05	0.02	0.003	1.02	1.08
NA staffing below ward mean	1.04	0.02	0.011	1.01	1.08
(full model includes random effects for ward, omitted for brevity) df 41, AIC 33803.89 BIC 34271.41					

Supplementary table g: Staffing below the mean during the first five days: hazard of death within 5 days

	Haz. Ratio	Std. Err.	p-value	95% Confidence Interval (CI)	
NEWS on admission	1.35	0.01	0.000	1.33	1.37
SHMI Risk score	1.96	0.04	0.000	1.88	2.04
Emergency	2.60	0.70	0.000	1.54	4.39
Admissions per RN>125% of ward mean	1.06	0.04	0.142	0.98	1.15
Admissions per NA>125% of ward mean	1.04	0.04	0.279	0.97	1.13
RN staffing below ward mean	1.08	0.02	0.001	1.03	1.13
NA staffing below ward mean	1.02	0.02	0.359	0.98	1.07
(full model includes random effects for ward, omitted for brevity) df 41, AIC20584.56 BIC 21038.67					

Supplementary table h: Staffing below the mean during the first five days: hazard of death – emergency admissions only

	Haz. Ratio	Std. Err.	p-value	95% Confidence Interval (CI)	
NEWS on admission	1.24	0.007	<0.001	1.23	1.25
SHMI Risk score	1.82	0.025	<0.001	1.77	1.87
Admissions per RN>125% of ward mean	1.05	0.023	0.019	1.01	1.10
Admissions per NA>125% of ward mean	1.00	0.022	0.820	0.95	1.04
RN staffing below ward mean	1.03	0.012	0.015	1.01	1.05
NA staffing below ward mean	1.04	0.012	0.001	1.02	1.07
(full model includes random effects for ward, omitted for brevity) df 40, AIC 60449.97, BIC 60918.79					

Supplementary table i: Staffing below the mean (all days of stay) – hazard of death

	Haz. Ratio	Std. Err.	p-value	95% Confidence Interval (CI)	
NEWS on admission	1.25	0.007	<0.001	1.23	1.26
SHMI Risk score	1.85	0.026	<0.001	1.80	1.90
Emergency	1.13	0.128	0.278	0.91	1.41
RN staffing below ward mean	1.01	0.003	0.013	1.00	1.01
NA staffing below ward mean	1.00	0.003	0.135	1.00	1.01
(full model includes random effects for ward, omitted for brevity) Df 39, AIC 61898.22, BIC 62360.76					

Supplementary table j: Staffing below the mean during the first five days + weekend admission: hazard of death (full model)

	Haz. Ratio	Std. Err.	p-value	95% Confidence Interval (CI)	
Weekend admission	1.06	0.035	0.096	0.99	1.13
NEWS on admission	1.24	0.007	<0.001	1.23	1.25
SHMI Risk score	1.82	0.025	<0.001	1.77	1.87
Emergency	1.10	0.123	0.416	0.88	1.37
Admissions per RN>125% of ward mean	1.05	0.023	0.027	1.01	1.09
Admissions per NA>125% of ward mean	1.00	0.022	0.897	0.96	1.04
RN staffing below ward mean	1.03	0.012	0.011	1.01	1.05
NA staffing below ward mean	1.04	0.012	<0.001	1.02	1.07
(full model includes random effects for ward, omitted for brevity) df 42, AIC 61889.12, BIC 62387.23					

Supplementary table k: Staffing below the mean during the first five days + weekend stay: hazard of death (full model)

	Haz. Ratio	Std. Err.	p-value	95% Confidence Interval (CI)	
Weekend stay	0.58	0.020	<0.001	0.54	0.62
NEWS on admission	1.24	0.007	<0.001	1.23	1.25
SHMI Risk score	1.84	0.025	<0.001	1.79	1.89
Emergency	1.19	0.133	0.125	0.95	1.48
Admissions per RN>125% of ward mean	1.03	0.022	0.152	0.99	1.08
Admissions per NA>125% of ward mean	0.99	0.021	0.517	0.95	1.03
RN staffing below ward mean	1.05	0.013	<0.001	1.03	1.08
NA staffing below ward mean	1.04	0.012	<0.001	1.02	1.07

(full model includes random effects for ward, omitted for brevity)
Df 42, AIC 61656.81, BIC 62154.93

Supplementary table l SHMI admission diagnostic groups in patients who died. Patient who died who were exposed to low staffing (RN or NA, below ward mean) on first day of admission (4377) vs Patient without low staffing exposure (1480)

SHMI Group	Exposed		Not exposed		All	
	n	%	n	%	n	%
Pneumonia	958	21.9%	257	17.4%	1215	20.7%
Acute cerebrovascular disease	376	8.6%	120	8.1%	496	8.5%
Congestive heart failure; nonhypertensive	210	4.8%	69	4.7%	279	4.8%
Septicaemia (except in labour); Shock	191	4.4%	60	4.1%	251	4.3%
Chronic obstructive pulmonary disease and bronchiectasis	129	3.0%	47	3.2%	176	3.0%
Acute and unspecified renal failure	126	2.9%	46	3.1%	172	2.9%
Urinary tract infections	123	2.8%	37	2.5%	160	2.7%
Acute myocardial infarction	105	2.4%	46	3.1%	151	2.6%
Cancer of bronchus; lung	98	2.2%	32	2.2%	130	2.2%
Gastrointestinal hemorrhage	93	2.1%	30	2.0%	123	2.1%
Aspiration pneumonitis; food/vomitus	85	1.9%	27	1.8%	112	1.9%
Fracture of neck of femur (hip)	69	1.6%	28	1.9%	97	1.7%
Respiratory failure; insufficiency; arrest (adult)	64	1.5%	28	1.9%	92	1.6%
Peripheral and visceral atherosclerosis	70	1.6%	18	1.2%	88	1.5%
Intestinal infection	61	1.4%	17	1.2%	78	1.3%
Acute bronchitis	66	1.5%	9	0.6%	75	1.3%
Secondary malignancies	44	1.0%	26	1.8%	70	1.2%
Other gastrointestinal disorders	49	1.1%	20	1.4%	69	1.2%
Pleurisy; pneumothorax; pulmonary collapse	54	1.2%	12	0.8%	66	1.1%
Other connective tissue disease	39	0.9%	14	0.9%	53	0.9%
Aortic; peripheral; and visceral artery aneurysms	42	1.0%	10	0.7%	52	0.9%
Pulmonary heart disease	39	0.9%	12	0.8%	51	0.9%
Cardiac arrest and ventricular fibrillation	31	0.7%	16	1.1%	47	0.8%
Liver disease; alcohol-related	35	0.8%	12	0.8%	47	0.8%
Biliary tract disease	39	0.9%	8	0.5%	47	0.8%
Other liver diseases	35	0.8%	10	0.7%	45	0.8%
Mental retardation; Senility and organic mental disorders	37	0.8%	8	0.5%	45	0.8%
Intestinal obstruction without hernia	36	0.8%	8	0.5%	44	0.8%
Fluid and electrolyte disorders	33	0.8%	10	0.7%	43	0.7%
Superficial injury; contusion	18	0.4%	20	1.4%	38	0.6%
Hodgkin’s disease; Non-Hodgkin’s lymphoma	24	0.5%	14	0.9%	38	0.6%
Cancer of bone, connective tissue, thyroid or NS	24	0.5%	13	0.9%	37	0.6%
Cancer of colon	28	0.6%	9	0.6%	37	0.6%
Leukemias	27	0.6%	9	0.6%	36	0.6%
Skin and subcutaneous tissue infections	28	0.6%	8	0.5%	36	0.6%
Deficiency and other anemia	26	0.6%	9	0.6%	35	0.6%
Cardiac dysrhythmias	28	0.6%	7	0.5%	35	0.6%
Intracranial injury	23	0.5%	11	0.7%	34	0.6%
Cancer of breast	24	0.5%	10	0.7%	34	0.6%
Cystic fibrosis; Other lower respiratory disease	25	0.6%	8	0.5%	33	0.6%
Influenza & other upper respiratory infections & disease	20	0.5%	12	0.8%	32	0.5%
Cancer of prostate or male genital organs	22	0.5%	10	0.7%	32	0.5%
Other fractures, sprains, strains, & joint disorders	13	0.3%	17	1.2%	30	0.5%
All others (n<30)	710	16.2%	286	19.3%	996	17.0%
**Total**	4377		1480		5857	

Staffing versus SHMI/NEWS

1. Staffing levels (RN or NA) were assigned to quartiles for each ward
2. SHMI (as raw logit) and NEWS values were extracted from the survival data for each of the first FIVE days of admission
3. Mean SHMI and NEWS values were calculated for each day on each ward
4. Three mixed-effects models were fit:
   a) Quartile ~ SHMI.Mean + NEWS.Mean + ward(as random effect)
   b) Above ward mean ~ SHMI.Mean + NEWS.Mean + ward(as random effect)
   c) Deviation from ward mean ~ SHMI.Mean + NEWS.Mean + ward(as random effect)

Supplementary table m: Association between SHMI risk, First NEWS and RN staffing (Quartile)

term	estimate	std.error	CI Lower	CI Upper
(Intercept)	2.51	0.0238	2.46	2.56
pas_shmi_score.mean	-0.00136	0.00383	-0.00887	0.00615
First.NEWS.mean	-0.00688	0.00639	-0.0194	0.00564

Supplementary table n: Association between SHMI risk, First NEWS and RN staffing (above mean)

term	estimate	std.error	p.value	CI Lower	CI Upper
(Intercept)	-0.11	0.0562	0.0492	-0.221	-0.000364
pas_shmi_score.mean	0.00831	0.0103	0.418	-0.0118	0.0284
First.NEWS.mean	0.000531	0.0133	0.968	-0.0255	0.0265

Supplementary table o: Association between SHMI risk, First NEWS and RN staffing (deviation from mean)

term	estimate	std.error	CI Lower	CI Upper
(Intercept)	-0.023	0.0184	-0.0591	0.013
pas_shmi_score.mean	-0.00485	0.00296	-0.0107	0.000957
First.NEWS.mean	-0.000317	0.00494	-0.01	0.00936

Supplementary table p: Association between SHMI risk, First NEWS and NA staffing (Quartile)

term	estimate	std.error	CI Lower	CI Upper
(Intercept)	2.51	0.0243	2.46	2.55
pas_shmi_score.mean	-0.00309	0.00394	-0.0108	0.00463
First.NEWS.mean	-0.00602	0.00647	-0.0187	0.00665

Supplementary table q: Association between SHMI risk, First NEWS and NA staffing (above mean)

term	estimate	std.error	p.value	CI Lower	CI Upper
(Intercept)	-0.118	0.0628	0.0597	-0.241	0.00483
pas_shmi_score.mean	0.00237	0.0114	0.835	-0.0199	0.0247
First.NEWS.mean	-0.00166	0.0136	0.903	-0.0283	0.025

Supplementary table r: Association between SHMI risk, First NEWS and NA staffing (deviation from mean)

term	estimate	std.error	CI Lower	CI Upper
(Intercept)	-0.00766	0.0185	-0.0439	0.0286
pas_shmi_score.mean	-0.00427	0.00321	-0.0106	0.00202
First.NEWS.mean	-0.00348	0.00453	-0.0124	0.00539

Supplementary table s: Cumulative sum of staffing hours per patient relative to the ward mean during the first five days: hazard of death

	Haz. Ratio	Std. Err.	p-value	95% Confidence Interval (CI)	
NEWS on admission	1.24	0.007	<0.001	1.22	1.25
SHMI Risk score	1.82	0.024	<0.001	1.77	1.87
Emergency	1.15	0.128	0.222	0.92	1.43
Admissions per RN>125% of ward mean	1.06	0.022	0.003	1.02	1.11
Admissions per NA>125% of ward mean	1.02	0.020	0.238	0.98	1.06
RN Hours	0.97	0.013	0.023	0.94	1.00
NA Hours	1.01	0.015	0.394	0.98	1.04
(full model includes random effects for ward, omitted for brevity) Df 41, AIC 61919.40, BIC 62405.66					

Supplementary table t: Cumulative sum of staffing hours per patient below the mean during the first five days: hazard of death

	Haz. Ratio	Std. Err.	p-value	95% Confidence Interval (CI)	
NEWS on admission	1.24	0.007	<0.001	1.22	1.25
SHMI Risk score	1.82	0.024	<0.001	1.77	1.87
Emergency	1.15	0.128	0.220	0.92	1.43
Admissions per RN>125% of ward mean	1.05	0.021	0.008	1.01	1.10
Admissions per NA>125% of ward mean	1.03	0.019	0.091	0.99	1.07
RN Hours below ward mean (cum sum)	1.03	0.016	0.046	1.00	1.06
NA Hours below ward mean (cum sum)	1.00	0.018	0.849	0.97	1.04
(full model includes random effects for ward, omitted for brevity) Df 41, AIC 61920.08, BIC 62406.33					

Supplementary table u: Cumulative sum of staffing hours per patient relative to the mean during the first five days: hazard of death including quadratic and cubic terms

	Haz. Ratio	Std. Err.	p-value	95% Confidence Interval (CI)	
NEWS on admission	1.24	0.007	<0.001	1.22	1.25
SHMI Risk score	1.82	0.024	<0.001	1.77	1.87
Emergency	1.15	0.128	0.216	0.92	1.43
Admissions per RN>125% of ward mean	1.06	0.022	0.004	1.02	1.10
Admissions per NA>125% of ward mean	1.02	0.020	0.265	0.98	1.06
RN Hours	0.98	0.015	0.200	0.95	1.01
RN Hours^2	1.00	0.003	0.621	0.99	1.01
RN Hours ^3	1.00	<0.001	0.930	1.00	1.00
NA Hours	1.00	0.020	0.835	0.97	1.04
NA Hours ^2	1.01	0.004	0.014	1.00	1.02
NA Hours ^3	1.00	0.001	0.061	1.00	1.00
(full model includes random effects for ward, omitted for brevity) Df 45, AIC 61920.19, BIC 62453.88					

Supplementary table v: Hazard of death associated with cumulative hours above or below mean during the first five days (with RN X NA interaction)

	Haz. Ratio	Std. Err.	p-value	95% Confidence Interval (CI)	
NEWS on admission	1.24	0.007	<0.001	1.22	1.25
SHMI Risk score	1.82	0.024	<0.001	1.77	1.87
Emergency	1.15	0.128	0.222	0.92	1.43
Admissions per RN>125% of ward mean	1.06	0.022	0.003	1.02	1.11
Admissions per NA>125% of ward mean	1.02	0.020	0.241	0.98	1.06
RN Hours	0.97	0.014	0.048	0.94	1.00
NA Hours	1.01	0.015	0.362	0.98	1.04
RN X NA Interaction	1.00	0.004	0.699	0.99	1.01
(full model includes random effects for ward, omitted for brevity)					

Seasonal Linear model (figures a,b)

Mixed-effects linear model of staffing ~ month + year + ward(as random effect). Plot shows fixed-effects coefficients for each month (June Mo 6 is reference month)

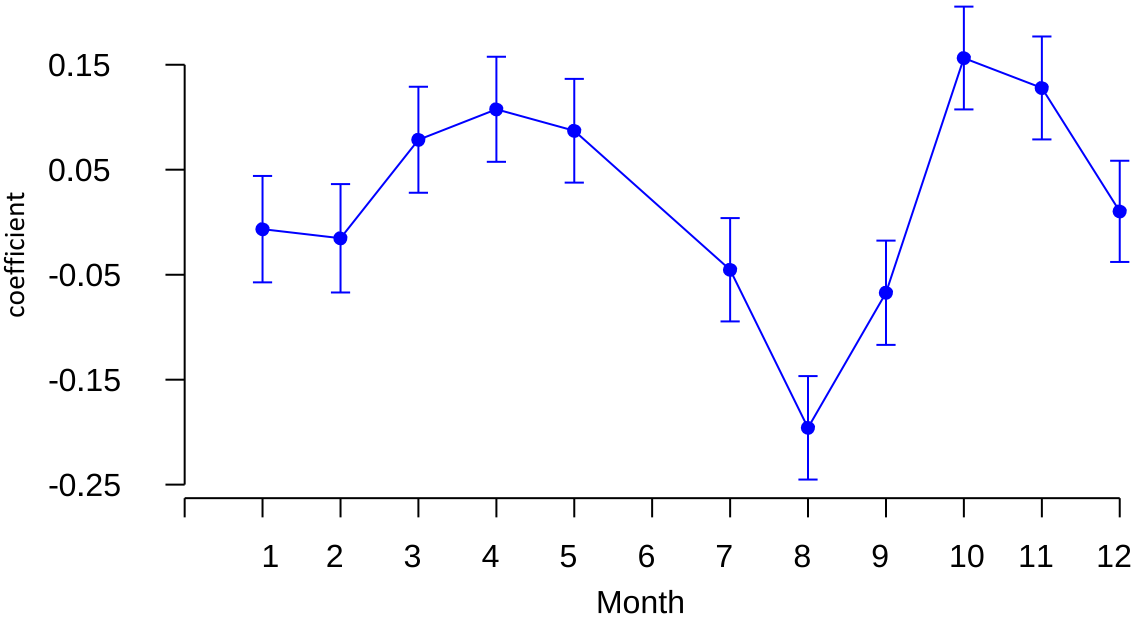

Supplementary figure a: Seasonal variation in RN staffing

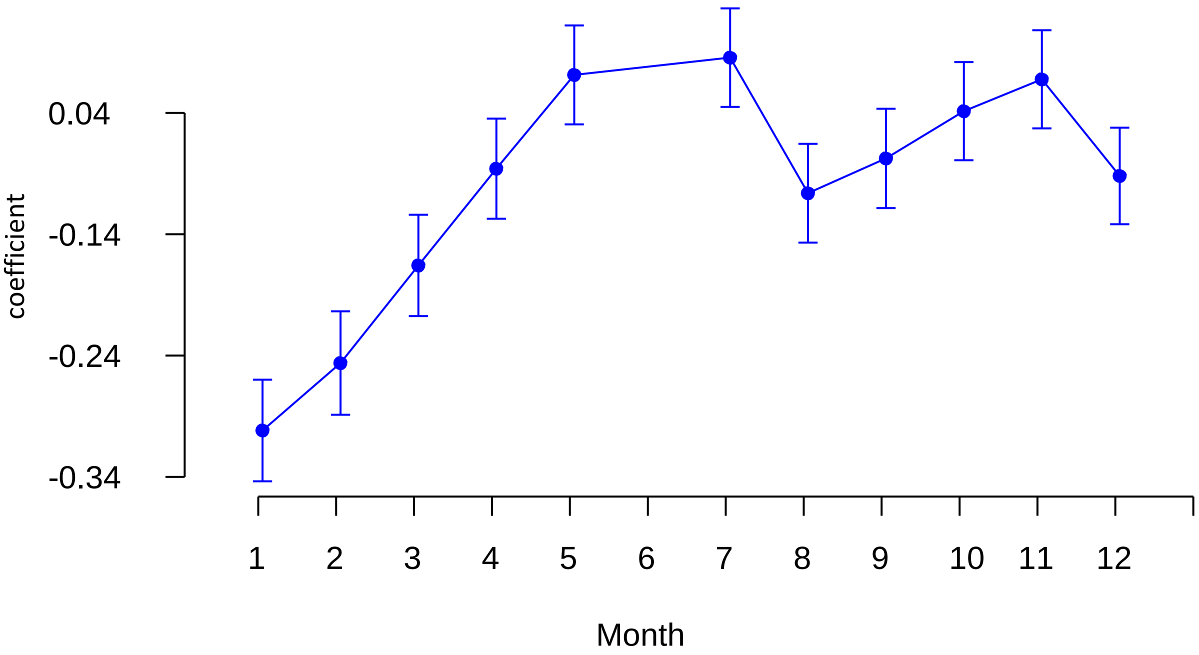

Supplementary figure b: Seasonal variation in NA staffing

1. The Shelford group. Safer Nursing Care Tool Implementation Resource Pack: The Shelford Group, 2014.
